# Detection of Endo-epicardial Asynchrony in the Atrial Wall Using One-Sided Unipolar and Bipolar Electrograms

**DOI:** 10.1007/s12265-021-10111-1

**Published:** 2021-03-29

**Authors:** Lisette J. M. E. van der Does, Roeliene Starreveld, Rohit K. Kharbanda, Paul Knops, Charles Kik, Ad J. J. C. Bogers, Natasja M. S. de Groot

**Affiliations:** 1 Department of Cardiology, Erasmus Medical Center, Dr. Molewaterplein 40, 3015GD, Rotterdam, Netherlands; 2 Department of Cardiothoracic Surgery, Erasmus Medical Center, Rotterdam, Netherlands

**Keywords:** Endo-epicardial asynchrony, Fractionated electrograms, Unipolar electrograms, Bipolar electrograms, Mapping

## Abstract

Endo-epicardial asynchrony (EEA) is a new mechanism possibly maintaining atrial fibrillation. We aimed to determine the sensitivity and best recording modus to detect EEA on electrograms recorded from one atrial side using electrogram fractionation. Simultaneously obtained right atrial endo- and epicardial electrograms from 22 patients demonstrating EEA were selected. Unipolar and (converted) bipolar electrograms were analyzed for presence and characteristics of fractionation corresponding to EEA. Sensitivity of presence of EEA corresponding fractionation was high in patients (86–96%) and moderately high (65–78%) for the asynchronous surface area for unipolar and bipolar electrograms equally. In bipolar electrograms, signal-to-noise ratio of EEA corresponding fractionation decreased and additional fractionation increased for electrograms recorded at the endocardium. Sensitivity of fractionation corresponding to EEA is high for both unipolar and bipolar electrograms. Unipolar electrograms are more suited for detection of EEA due to a larger signal-to-noise ratio and less disturbance of additional fractionation.

**Graphical Abstract:**

**Figure:**
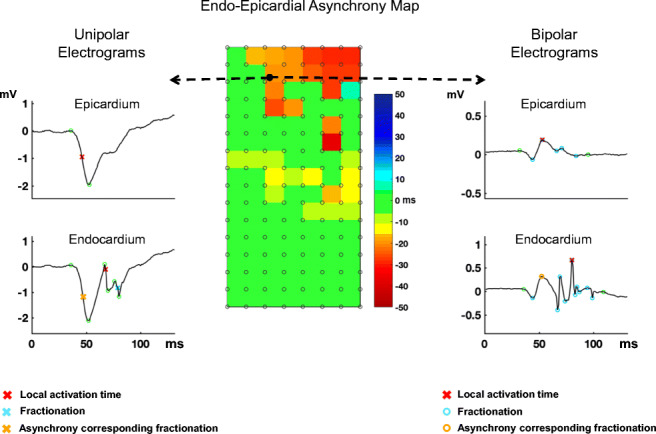
Unipolar electrograms are more suited than bipolar electrograms to detect endo-epicardial asynchrony on one side of the atrial wall using electrogram fractionation.

**Supplementary Information:**

The online version contains supplementary material available at 10.1007/s12265-021-10111-1.

## Introduction

The electrical pathophysiological mechanisms of persistent atrial fibrillation remain to this day largely unknown. Recent evidence suggests that dissociated electrical conduction between the layers of the atrial wall presenting as endo-epicardial asynchrony (EEA) in excitation is a potential significant mechanism for persistence of atrial fibrillation [1, 2]. The asynchronous activation of epicardial and endocardial layers provides opportunity for waves of excitation to travel transmurally and cause new breakthrough waves on the opposite side of the wall. After canine and goat models, a new simultaneous endo-epicardial mapping approach finally allowed for documentation of EEA of the right atrial wall in patients as well [1, 3, 4]. However, this technique can only be applied in patients undergoing cardiac surgery and is limited to the right atrial appendage/free wall and occasionally the left atrial appendage [5]. A method to detect EEA during endovascular electrophysiological studies would greatly benefit research into the mechanisms of atrial fibrillation. Recently, we investigated simultaneously recorded unipolar endocardial and epicardial electrograms during sinus rhythm and discovered that EEA causes unipolar electrogram fractionation (additional deflections on the electrogram). By relating unipolar electrogram fractionation to spatial patterns of activation, fractionation could be attributed to EEA [6]. However, most electrophysiological studies use a bipolar recording mode for mapping to reduce farfield effects recorded by unipolar electrograms [7]. In the case of EEA, remote activation on unipolar electrograms could be an important feature to detect EEA while recording on only one side of the atrial wall. We therefore hypothesized that unipolar electrograms are more sensitive in detection of atrial EEA than bipolar electrograms. Electrogram features of sites with EEA were analyzed in 22 patients and we compared the sensitivity of unipolar and bipolar electrograms for detection of EEA from only one side of the atrial wall.

## Methods

### Study Population

Twenty-two patients from the ongoing Epic End study in the Erasmus Medical Center were selected. The Epic End study is approved by the local medical ethics committee (MEC-2015-373) and includes patients over 18 years of age undergoing cardiac surgery for coronary artery disease, heart valve disease, and/or congenital heart disease. This study complies with the Declaration of Helsinki and prior to participation all patients gave informed consent. Mean age of selected patients was 65±9 years and 15 of 22 were male. Cardiac surgery was performed for coronary artery disease (*N*=15) and/or valvular heart disease (*N*=12); ten patients had a history of atrial fibrillation of whom one had persistent atrial fibrillation.

### Endo-epicardial Mapping

**Fig. 1:**
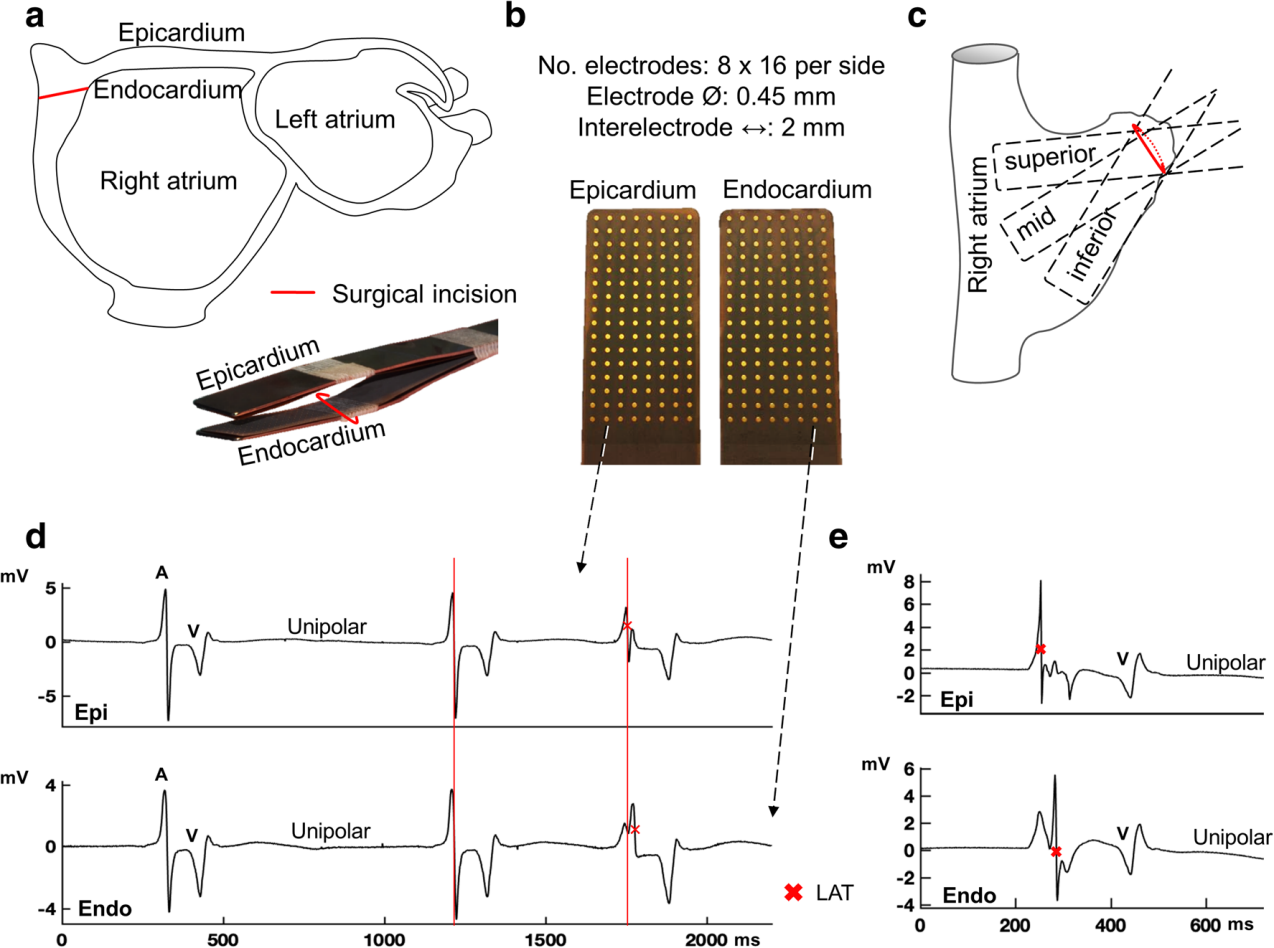
Simultaneous endo-epicardial mapping in patients during cardiac surgery. **a** The mapping tool consists of two identical electrode arrays fixed to each other. One leg (electrode array) of the mapping tool is introduced in the right atrium via a standard surgical incision for cardio-pulmonary bypass. This allows to record electrograms from the epicardium (outside wall) and endocardium (inside wall) simultaneously. **b** The properties of the electrode array. **c** Mapping locations at the right atrium. **d** Examples of directly opposite epicardial (epi) and endocardial (endo) unipolar electrograms. Endo-epicardial atrial activation (A) is in synchrony in the first two beats, the following atrial extrasystole demonstrates asynchronous endo-epicardial atrial activation. V, ventricular activation; LAT, local activation time. **e** Example of atrial asynchrony and additional deflections next to the LAT deflection on unipolar electrograms (=fractionation). One fractionation-deflection on each electrogram corresponds to the asynchronous activation on the opposite side

Mapping during cardiac surgery was performed just prior to commencement of cardiopulmonary bypass and after arterial cannulation. Simultaneous endo-epicardial mapping was conducted by introducing one of two 128-electrode (8×16) arrays in the right atrium via the incision for venous cannulation for endocardial mapping. The other array was placed on top of the epicardium for epicardial mapping. Both electrode arrays (0.45-mm electrodes, 2-mm interelectrode spacing) were fixed on a steel spatula and bound together to ensure good contact and precise alignment of the two arrays. An indifferent electrode is attached to a steel wire fixed in subcutaneous tissue and a reference signal is stitched to the right atrium. Unipolar electrograms of the right atrial wall were recorded for 5–10 s during sinus rhythm and pacing at the superior, middle, and inferior right atrial free wall (Fig. 1A–C). In one patient, endo-epicardial electrograms were recorded from the left atrial appendage before excision. Electrograms were sampled at 1000 Hz, filtered (0.5–400 Hz) and digitized (16-bits conversion) and, with a calibration signal of 2 mV, stored on hard disk for offline analysis. Details of the endo-epicardial mapping procedure were previously described [5].

### Data Analysis

#### Electrogram Selection, Conversion, and Marking

Recorded data during sinus rhythm and pacing of all patients included in our study were analyzed for the presence of EEA (see Fig. 1D). Only patients demonstrating EEA were included and if multiple recording sites of a patient demonstrated EEA, only the recording site with the largest area of EEA was included. Local activation time (LAT) in unipolar electrograms was marked at the steepest negative slope (dV/dt) with a minimum of 0.05 mV/ms. Activation maps were constructed for both epicardium and endocardium. EEA was determined from these maps by calculating the differences between the local activation time at each electrode and the 9 opposite electrodes in the other plane: direct opposite and its 8 surrounding electrodes. Minimal time difference with these 9 opposite electrode sites determined the time difference for the electrode. EEA was defined as a difference between *epi*cardial and *endo*cardial local activation time of ≥15ms. If unipolar asynchrony maps demonstrated EEA at ≥4 adjacent electrode sites that did not include border electrodes, the recording site was included for analysis. Border electrodes, defined as electrodes with <7 opposite local activation times, and electrodes missing the exact opposite electrogram were excluded from analysis. One electrode site corresponds to an area of 4mm^2^.

**Fig. 2:**
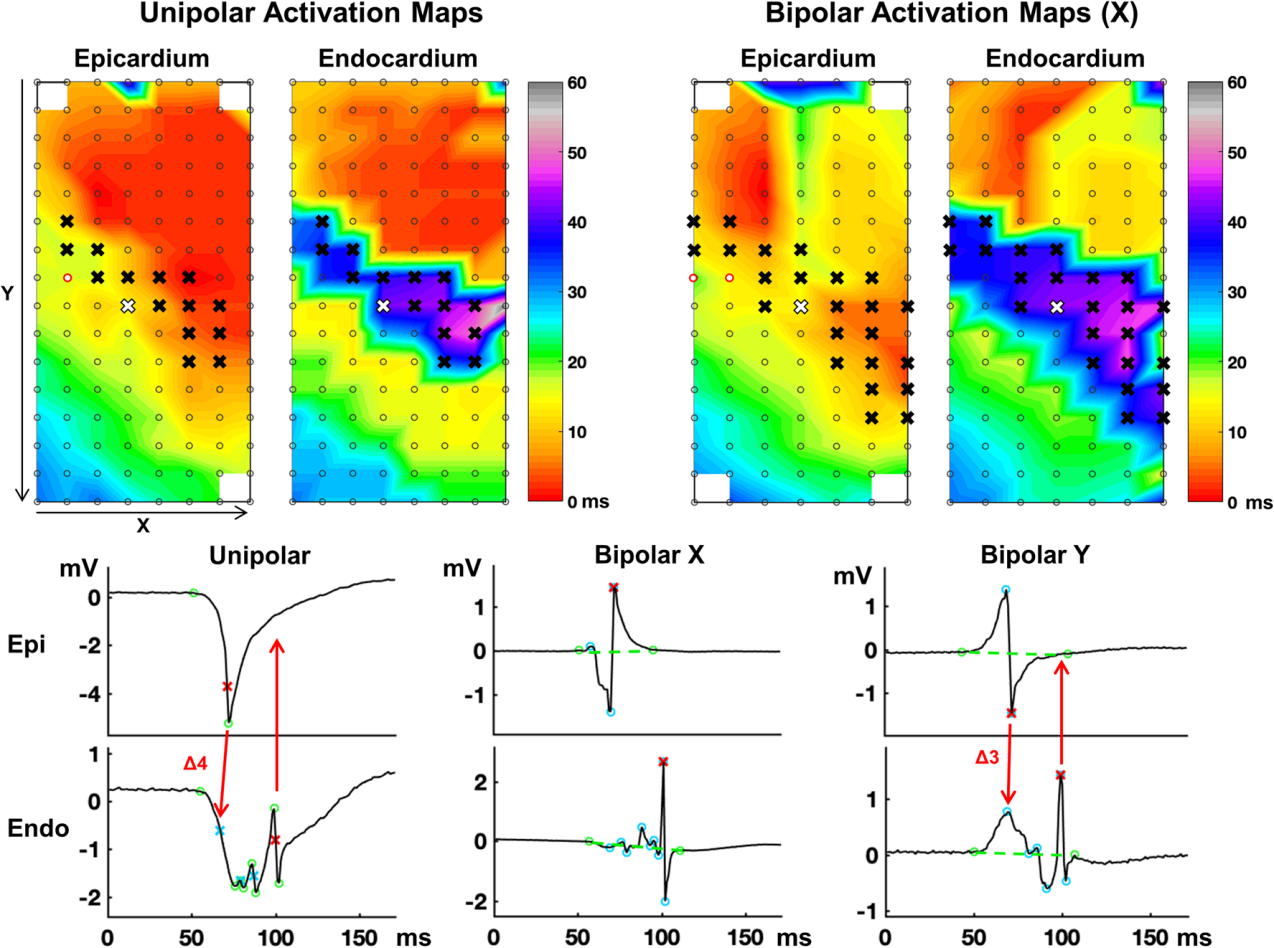
Data selection and analysis. Top left: epi- and endocardial activation maps constructed from unipolar electrograms acquired from simultaneous endo-epicardial mapping. Top right: endo-epicardial activation maps constructed after subtracting unipolar electrograms in the horizontal (*X*) direction creating bipolar electrograms. Crosses indicate electrogram sites with endo-epicardial asynchrony (EEA) that are included for the study. Red circles represent a broken electrode site and endo-epicardial electrograms at this site are excluded from the study. Bottom: unipolar and bipolar epi- and endocardial electrograms from the site marked with the white cross. Local activation time is marked at the steepest slope for unipolar electrograms and at the largest peak for bipolar electrograms (red crosses). Fractionation time (FT) is determined by marking the steepest slope of additional deflections for unipolar electrograms and by marking additional peaks for bipolar electrograms (blue markers). If the difference between a FT and the local activation time of the opposite electrogram is ≤7ms, 4ms in the unipolar electrogram example and 3ms in the bipolar (Y) electrogram example, this fractionation is defined as fractionation corresponding to EEA. In this example, no fractionation corresponding to EEA (the endocardial local activation time) is present on the epicardial electrogram. Unipolar voltage of corresponding fractionation is measured as the difference between peaks (between green circles). Bipolar voltage of corresponding fractionation is measured as the difference between peak and baseline; baseline is virtually constructed as a straight line (green line) between the two green markers placed by the observers in bipolar electrograms (green circles) thereby correcting for baseline drift. Bipolar fractionation (blue circled peaks) within noise level of this virtual baseline is excluded from analysis; in this example, the second blue circled peak on the *y*-bipolar endocardial electrogram is excluded. Fractionation (blue crosses or peaks) that does not correspond to EEA with a difference ≥15ms of the local activation time in the same electrogram is counted to determine presence of additional fractionation

Unipolar electrograms were converted to bipolar electrograms by subtracting the unipolar electrogram from one electrode from the unipolar electrogram at the adjacent electrode of the array. Bipolar conversion was performed two times: in the horizontal (*x*) direction and in the vertical (*y*) direction. Local AT in bipolar electrograms was marked at the largest (maximal or minimal) peak. EEA and electrode inclusion was then determined as described above with the exception that bipolar electrograms at the right or left border in case of *x*-direction conversion and top or bottom border in case of *y*-direction conversion were included for analysis (Fig. 2). In addition, for the bipolar activation maps, only electrodes with EEA on similar sites of EEA on the unipolar activation maps were included. This assured only electrograms from the same EEA site were analyzed so there was no disagreement between the unipolar and bipolar EEA sites.

Included unipolar electrograms with EEA were then inspected for visual presence of additional (fractionated/farfield) deflections (Fig. 1E), or additional peaks in case of bipolar electrograms. All markings were evaluated by two investigators independently. Bipolar fractionation peaks were marked by the investigators based on previous studies marking bipolar fractionated electrograms using the change in polarity of the depolarization slope to tag bipolar peaks [8, 9]. Each additional marked peak on bipolar electrograms within baseline noise, defined as up to 120% of the noise, was excluded. Of each primary (=LAT) and fractionated unipolar deflection, the following parameters were derived: amplitude (peak-to-peak voltage), the time of steepest slope (FT, fractionation time), and signal-to-noise ratio (SNR). Primary and fractionation peaks of bipolar electrograms were analyzed for voltage (peak-to-baseline), time of the peaks (LAT or FT), and SNR.

#### Corresponding Fractionation Analysis

At each EEA site, the primary *epi*cardial deflection or peak and the *endo*cardial primary deflection or peak were compared to the direct opposite electrogram for the presence of fractionation corresponding to the primary deflection/peak. If the FT of a fractionation peak or deflection on the opposite side was ≤7 ms of the LAT, it was labeled as corresponding fractionation (to the primary deflection/ peak) (Fig. 2). This cut-off was chosen based on our previous definitions of conduction delay and block [10]. In case of multiple deflections or peaks meeting this criterion, first the closest deflection/peak, otherwise the largest deflection/peak, was selected as fractionation corresponding to EEA. Parameters of the corresponding unipolar deflection or bipolar peak included voltage, SNR and voltage compared to the primary deflection/peak on the same electrogram (in %). In addition, the time difference between the LAT and corresponding FT was analyzed to determine level of time accuracy.

#### Additional Fractionation

Besides analysis of fractionation corresponding to EEA, each opposite electrogram was analyzed for the presence of fractionation in addition to the EEA corresponding fractionation with a FT ≥15 ms separated from the LAT. This fractionation can be confused for EEA and does not correspond to the primary deflection on the other side and could complicate determining presence of EEA.

#### Statistical Analysis

Data with a normal distribution are presented as the mean ±SD and skewed data are presented as the median (p25–p75). To assess differences between unipolar and bipolar electrograms, Friedman’s test was used in case of skewed data and ANOVA repeated measures was used in case of normally distributed data. Post hoc tests between (1) unipolar and bipolar-*x* and (2) unipolar and bipolar-*y* were performed with Wilcoxon signed rank test. Statistical significance was set at *p*≤0.05, and post hoc test significance levels were adjusted according to Bonferroni at *p*≤0.025. The Mann-Whitney *U* test was used for comparison between patients with different recording sites and atrial rhythms. Data of which outcomes of statistical significance were similar between observers is presented in the text as the mean of the two medians and percentiles and the highest *p*-value is presented.

## Results

### EEA Area and Electrogram Characteristics

Included EEA areas occurred during sinus rhythm in 14 patients, during an atrial extrasystole in 7 patients and during pacing at 240 bpm in one patient. Most recordings were from the superior right atrial wall (*n*=14), followed by the mid-right atrial wall (*n*=6) (Supplemental Table 1). EEA was present on a median surface of 52 (31–94) mm^2^ in unipolar maps and no difference was observed between unipolar and bipolar maps (bipolar-*x*: 42 (22–87) mm^2^, bipolar-*y*: 52 (32–94) mm^2^, *p*=0.78, see Table 1). Activation time differences between epicardium and endocardium in unipolar maps ranged from 16 to 96 ms per patient with a median delay of 26 (21–33) ms. Bipolar endo-epicardial delays were similar to unipolar endo-epicardial delays (*p*=0.37). Amplitudes of bipolar electrograms were lower compared to unipolar electrograms for both epicardial and endocardial electrograms (*p*<0.001). In addition, SNR of bipolar electrograms in the *y*-direction was lower compared to unipolar electrograms: 32 (16–62) vs 62 (32–114) for *epi*cardial electrograms and 13 (5–35) vs 28 (18–52) for *endo*cardial electrograms (*p*≤0.001).

**Table 1:** Endo-epicardial asynchrony and electrogram characteristics

	Unipolar		Bipolar-*x*		Bipolar-*y*		*p*-value
EEA area (mm^2^)	52	(31–94)	42	(22–87)	52	(32–94)	0.78
EEA delay (ms)	26	(21–33)	25	(22–32)	24	(21–30)	0.37
Epicardium							
Voltage* (mV)	3.4	(2.1–5.9)	1.1	(0.7–3.0)	1.4	(0.9–2.9)	<0.001
SNR	62	(32–114)	73	(25–115)	32	(16–62)	<0.001
Endocardium							
Voltage* (mV)	1.8	(1.1–2.8)	0.5	(0.3–1.3)	0.8	(0.2–1.8)	<0.001
SNR	28	(18–52)	29	(7–52)	13	(5–35)	<0.001

*Maximal peak-to-peak voltage of uni- and bipolar electrograms

*EEA*, endo-epicardial asynchrony; *SNR*, signal-to-noise ratio

### EEA Corresponding Fractionation

**Fig. 3:**
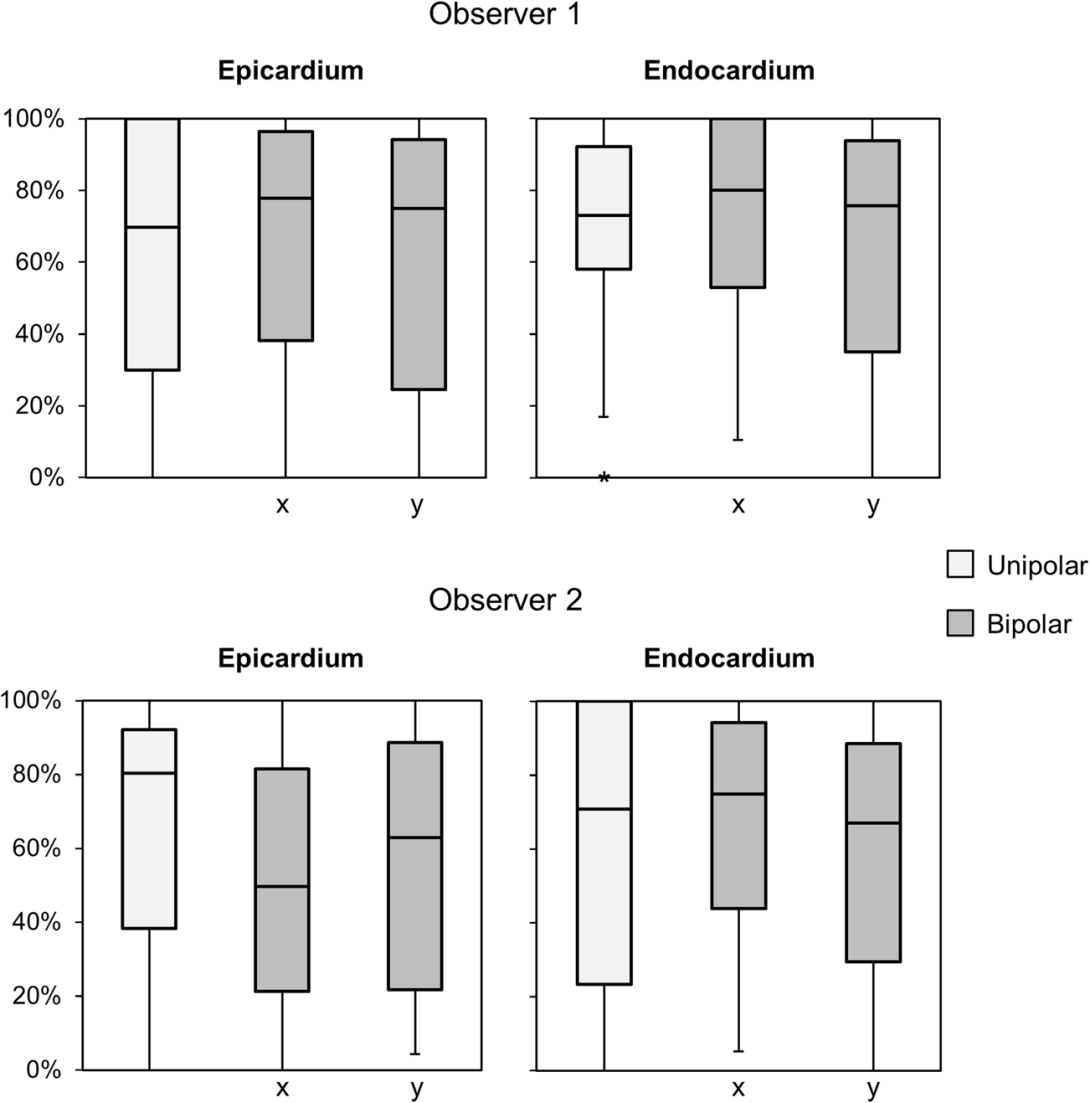
Presence of EEA corresponding fractionation on unipolar and bipolar electrograms. Boxplots of the percentage of electrode sites with EEA where the electrogram shows fractionation corresponding to EEA for observer 1 (top) and observer 2 (bottom). Outliers (>1.5 interquartile range) are presented as asterisks

Both unipolar and bipolar electrograms demonstrated EEA-related fractionation in equal amounts (see Fig. 3). Fractionation corresponding to EEA was present at 75% (34–96%) of the electrode sites per patient for *epi*cardial unipolar electrograms and at 72% (41–96%) for *endo*cardial unipolar electrograms. Bipolar *epi*cardial electrograms showed fractionation corresponding to EEA at 64% (30–89%) of electrode sites per patient in the *x*-direction and 69% (24–92%) in the *y*-direction. Bipolar *endo*cardial electrograms showed EEA corresponding fractionation in the *x*- and *y*-direction at respectively 78% (49–97%) and 72% (33–92%) of electrode sites. Complete absence of EEA fractionation occurred in one patient (<5%) for unipolar *epi*cardial electrograms and in maximal two patients (<10%) for bipolar *epi*cardial electrograms and in maximal three patients (<14%) for unipolar and bipolar *endo*cardial electrograms (Supplemental Table 2).

**Fig. 4:**
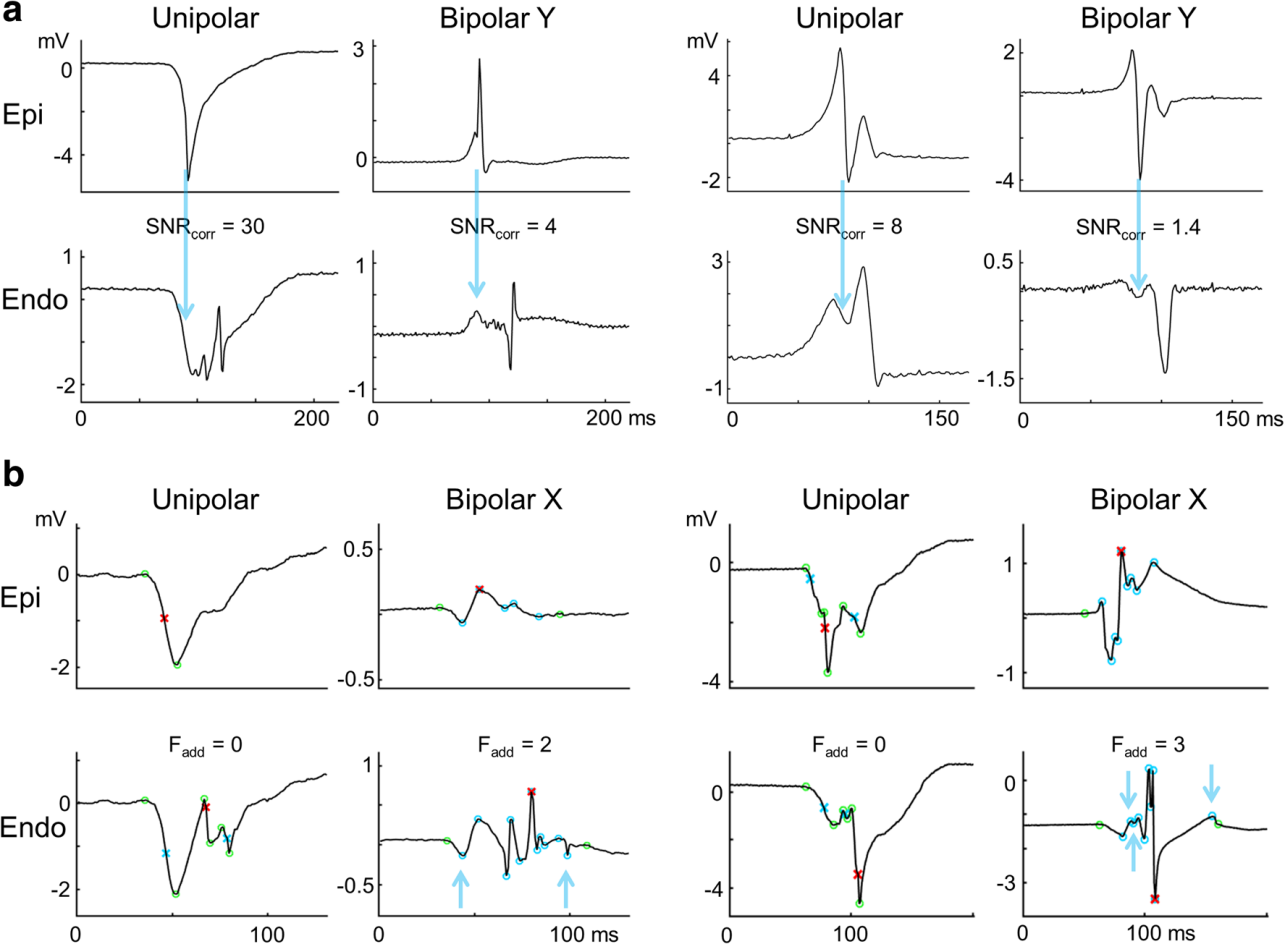
Unipolar versus bipolar electrograms. **a** Two electrogram examples demonstrating decrease of signal-to-noise ratio of fractionation corresponding to EEA (SNR_corr_) in bipolar endocardial electrograms in the *y*-direction. Blue arrow points to fractionation on the endocardial electrogram corresponding to the primary deflection/peak of the local activation time on the epicardial electrogram. **b** Two electrogram examples demonstrating increase of additional fractionation (*F*_add_) on the bipolar endocardial electrogram in the *x*-direction. Red cross indicates local activation time. Blue crosses or circles indicate fractionation. Blue arrows indicate fractionation which (1) does not correspond to EEA, (2) not within noise level of the baseline, and (3) is ≥15ms removed from the local activation time (= *F*_add_)

Absolute voltage of EEA corresponding fractionation was higher on unipolar electrograms than on bipolar electrograms (Table 2). However, relative size of EEA corresponding fractionation to the primary deflection or peak, representing the LAT, did not differ between unipolar and bipolar electrograms. The SNR of corresponding fractionation, or the ease in which the signal can be separated from the noise, was significantly decreased in bipolar electrograms created in the *y*-direction at the *endo*cardium (unipolar SNR: 11 (6–25) vs bipolar-y SNR: 4 (2–7), *p*<0.001). Examples of SNR decrease in bipolar electrograms are shown in Fig. 4A. Time accuracy of corresponding FT compared to the LAT was similar for unipolar and bipolar electrograms at an average of 2 to 3 ms.

**Table 2:** Characteristics of corresponding fractionation

	Observer 1							Observer 2						
	Unipolar		Bipolar-*x*		Bipolar-*y*		*p*-value	Unipolar		Bipolar-*x*		Bipolar-*y*		*p*-value
Epicardium														
Voltage (mV)	0.61	(0.27–1.38)	0.19	(0.08–0.31)	0.28	(0.14–0.48)	<0.001	0.57	(0.21–1.35)	0.22	(0.12–0.31)	0.31	(0.17–0.48)	<0.001
Relative to primary (%)	24	(13–37)	17	(9–31)	21	(16–32)	0.51	20	(12–43)	20	(11–39)	26	(17–42)	0.49
SNR	13	(4–22)	10	(4–13)	5	(3–12)	0.04	11	(4–23)	12	(5–16)	5	(4–11)	0.12
Time accuracy (ms)	2	±1.8	3	±1.3	3	±1.3	0.56	3	±1.6	3	±1.5	3	±1.6	0.60
Endocardium														
Voltage (mV)	0.56	(0.29–1.6)	0.17	(0.07–0.36)	0.18	(0.12–0.48)	<0.001	0.66	(0.27–1.74)	0.20	(0.09–0.37)	0.20	(0.11–0.45)	<0.001
Relative to primary (%)	49	(31–78)	34	(29–49)	34	(19–48)	0.14	50	(35–78)	39	(30–58)	35	(25–59)	0.37
SNR	11	(6–23)	9	(4–15)	4	(2–7)	<0.001	10	(6–26)	11	(3–18)	4	(2–7)	<0.001
Time accuracy (ms)	3	±1.6	2	±1.4	3	±1.1	0.53	3	±1.5	2	±1.6	3	±1.3	0.67

*SNR*, signal-to-noise ratio

### Additional Fractionation

The presence of other fractionation that does not correspond to EEA will complicate determining the presence of EEA based on fractionation. Table 3 presents the percentage of electrograms that showed fractionation other than the EEA corresponding fractionation and the average number of additional deflections/peaks per electrogram. At the endocardium, bipolar electrograms in the *x*-direction demonstrated more additional fractionation than unipolar electrograms: 82% (52–100) vs 53% (10–86) (*p*=0.019) of electrograms and 2 peaks (1–3) vs 1 (0–1) deflection per electrogram (*p*=0.004). Figure 4B shows examples of increase of additional fractionation on bipolar *endo*cardial electrograms.

**Table 3:** Presence of additional fractionation

	Unipolar		Bipolar-*x*		Bipolar-*y*		*p*-value
Epicardium							
Electrogram %							
Obs. 1	39	(0–79)	63	(24–100)	65	(29–87)	0.011
Obs. 2	48	(2–66)	49	(19–100)	60	(23–77)	0.098
No. per electrogram							
Obs. 1	0	(0–1)	1	(0–2)	1	(0–1)	0.002
Obs. 2	1	(0–1)	1	(0–2)	1	(1–1)	0.056
Endocardium							
Electrogram %							
Obs. 1	59	(11–90)	85	(55–100)	68	(37–87)	0.006
Obs. 2	46	(8–81)	79	(49–100)	63	(45–83)	0.002
No. per electrogram							
Obs. 1	1	(0–1)	2	(1–2)	1	(0–2)	0.002
Obs. 2	1	(0–1)	2	(1–3)	1	(1–1)	0.001

### Effect of Recording Rhythm and Site

In this small sample size, no differences in results were observed between the sinus rhythm and atrial extrasystole/pacing recordings. Only the endocardial bi-*x* electrograms of patients with recordings at the superior right atrium demonstrated more additional deflections compared to the other atrial regions combined (2.5 (2–3) vs 0.5 (0–2), *p*=0.040); other results between these two patient groups were similar.

### Interobserver Differences

Results of each observer are shown in Supplemental tables 2–6. Differences between observers in the significant statistical outcomes of the presented results above included SNR of corresponding fractionation at the *epi*cardium (*p*=0.04 vs *p*=0.12) and percentage and number of additional fractionation at the *epi*cardium (*p*=0.011 vs *p*=0.098 and *p*=0.002 vs *p*=0.056). At the endocardium, the higher number of additional fractionation per electrogram reached significance in only one observer (*p*=0.023 vs *p*=0.027).

## Discussion

Previously, it was shown that most fractionation occurs due to inhomogeneous conduction patterns [6, 11]. Almost all fractionated deflections in unipolar electrograms can be traced to neighboring electrical activation sites including the opposite side of the atrial wall [6]. Most clinical studies that have investigated electrogram fractionation use bipolar electrograms as this is the preferred recording method in clinical practice [7]. This study has demonstrated that EEA is reflected equally on unipolar and bipolar electrograms. However, fractionation reflecting EEA is less easy to distinguish from noise on endocardial electrograms using the bipolar recording mode. Furthermore, bipolar electrograms from the endocardium demonstrate more additional fractionation compared to unipolar electrograms that could complicate detection of EEA. This study has also shown that EEA reflects well on electrograms, over 86% of patients have at least one site showing fractionation corresponding to EEA.

### Factors Influencing Bipolar Electrograms

Because a bipolar electrogram is the product of two unipolar electrograms, several factors influence the morphology of a bipolar electrogram. For one, the distance between the two poles of a bipolar electrogram affects degree of fractionation. A computer model, which was also validated in a clinical population of atrial fibrillation patients, demonstrated that a larger interelectrode distance increases electrogram fractionation in bipolar electrograms in case of inhomogeneous activation patterns [12]. Also, increasing electrode size increases fractionation on both bipolar and unipolar electrograms [12]. Recordings of bipolar electrograms at scarred ventricular tissue representing a potential arrhythmogenic substrate in a study of Takigawa et al. confirmed the effect of orientation of the two poles on bipolar electrogram voltage and presence of abnormal electrograms of low voltage or with fractionation [13]. A parallel or transversal orientation of bipolar poles to the direction of activation resulted in differences in bipolar voltage of 50%. Sites with abnormal (fractionated) electrograms only matched in 57% between the different bipolar pole orientations and 30% of sites with abnormal (fractionated) electrograms were missed in the other pole orientation [13]. Therefore, the diverse morphology of bipolar electrograms based on electrode size, interelectrode spacing, and catheter orientation, especially under conditions of complex activation patterns, complicates the use of bipolar electrogram morphology.

### What Do Components of Fractionated Bipolar Electrograms Depict?

**Fig. 5:**
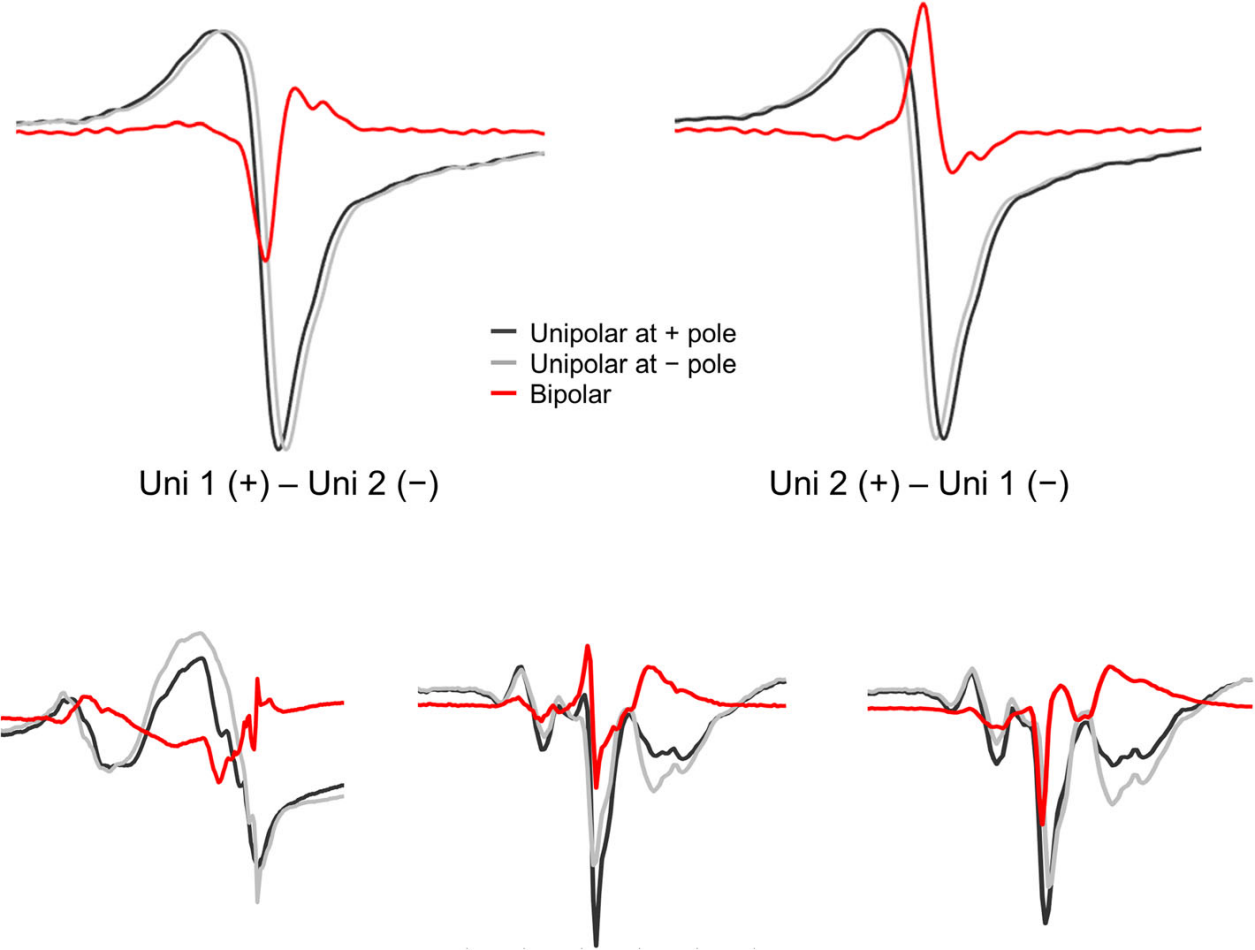
Peaks in bipolar electrograms Top left: a bipolar electrogram from two similar shaped signals with only one moved 1 sample on the time (*x*-)axis is the same as the derivative (∆) of the signal. The minimum of the derivative is the steepest negative slope of the original signal. Top right: if the positive and negative poles are switched, the bipolar electrogram is the negative derivate (−∆). The maximum of the bipolar electrogram is in this case the steepest negative slope of the signal. Bottom: three examples of fractionated unipolar electrograms where the two unipolar electrograms switch in which is de leading electrogram between the different fractionated components. For example, for the electrograms on the left, the light grey unipolar electrogram deflects negatively before the dark unipolar electrogram. The bipolar electrogram shows a positive peak at this point. However, with the following deflection, the dark unipolar electrogram deflects before the lighter unipolar electrogram deflects. Here, the bipolar electrogram shows a negative peak. Therefore, it is not possible in these bipolar electrograms to determine if a peak is a unipolar negative deflection (voltage decrease) or a rise (voltage increase) in the unipolar electrogram

Components (deflections) of a unipolar fractionated electrogram relate to remote parts of dissociatively activated myocardium, e.g., after a line of conduction block or to dissociation in activation of myocardial bundles underneath the electrode [11, 14]. A bipolar electrogram is meant to present (an approximation to) the derivate of the unipolar electrograms and the maximal peak in the derivate (or bipolar electrogram) coincides with the negative steepest slope(s) of the unipolar electrogam. The timing of the two unipolar signals (signal at the negative pole is earlier vs later than the signal at the positive pole) determines if the peak on the bipolar electrogram is a maximum or a minimum (top of Fig. 5). However, as seen in Fig. 5, the bipolar electrogram also demonstrates peaks for the (steepest) positive slopes of the unipolar electrograms. Converting fractionated unipolar electrograms to bipolar electrograms makes distinguishing between bipolar peaks due to positive or negative components of the unipolar electrograms impossible. The electrograms at the bottom of Fig. 5 demonstrate that peaks in bipolar electrograms can represent negative deflections as well as positive deflections in unipolar electrograms. This concept could explain why additional fractionation presented more frequently in bipolar electrograms in this study.

### EEA Detection with Use of Unipolar Fractionation

EEA has been suggested as a pathophysiological mechanism for persistence of atrial fibrillation [1]. Unfortunately, simultaneous mapping of epi- and endocardium is mostly limited to the right atrial free wall and only possible during cardiac surgery. Therefore, new techniques to identify EEA need to be developed in order to diversify research into the role of endo-epicardial asynchrony in arrhythmogenesis. Previously, we discovered that at least 95% of unipolar fractionation corresponds to remote activation by using automated detection of fractionation. In this study, fractionation was identified visually by two investigators to maximize detection of EEA-based fractionation and because automated signal detection in clinical practice is often evaluated by visual standards of the electrophysiologist. Outcome differences between the investigators were mainly limited to the epicardium. This may be explained by the larger SNR at the epicardium, making small peaks or deflections harder to detect visually. A positive finding is that a great majority (86%) of patients with EEA demonstrates fractionation corresponding to EEA on the other side of the atrial wall. This study did show that unipolar electrograms are better suited than bipolar electrograms for fractionation-based EEA detection due to less interference of additional fractionation and because EEA corresponding signals are better distinguishable from the noise. During atrial fibrillation, activation waves are often much smaller and with more complex activation patterns with frequent wave break, wave collision, and conduction block [15]. Unipolar voltage and SNR of fractionated components will be even smaller during atrial fibrillation than in this study emphasizing the use of unipolar over bipolar electrograms. The next steps in order to develop an EEA detection tool would be to (1) label unipolar fractionated deflections corresponding to remote activation in the longitudinal plane and (2) find the most sensitive and specific signal parameters to diagnose fractionated deflections corresponding to asynchronous activation within the atrial wall.

This kind of detailed marking of atrial electrograms with the fractionated deflections included will require automated software that is able to adjust marking settings to each recording accordingly and may require machine learning–based software development. Animal studies can help clarify fractionation occurrence based on *intra*mural asynchrony and thereby the specificity of EEA-based fractionation. Furthermore, in order to make the tool suitable for endovascular procedures, endovascular mapping would need technological advancements as well. Endovascular mapping will require high-density mapping arrays in order to detect neighboring sources of dissociated activation that can also have good wall contact as sensitivity could otherwise decrease compared to our study. If, finally, we will be able to accurately identify presence of asynchrony during arrhythmia, we will have better understanding of causes of ablation failures and possible solutions, and, in case of atrial fibrillation, could identify those patients in which the atrial myocardium had become so dissociated that any additional ablation is pointless. We are only at the start of the race and just took our first hurdle with this exploratory study.

### Study Limitations

Endo-epicardial mapping was mainly performed at the right atrial free wall as left atrial simultaneous endo-epicardial mapping can only be performed in very select cases. The differences between unipolar and bipolar electrograms may not apply to the thinner wall of the left atrium although an increase in sensitivity of asynchrony-based fractionation is expected at the left atrium compared to the right atrial free wall due to its thinner structure.

## Supplementary Information

ESM 1 (DOCX 35 kb).

## Abbreviations

EEA: endo-epicardial asynchrony
FT: fractionation time
LAT: local activation time
SNR: signal-to-noise ratio
