# Supplementary material for: Detection of Endo-epicardial Asynchrony in the Atrial Wall Using One-Sided Unipolar and Bipolar Electrograms
Source: J Cardiovasc Transl Res. 2021 Mar 29;14(5):902–11. doi: 10.1007/s12265-021-10111-1 (PMC8575733; doi:10.1007/s12265-021-10111-1)
Supplement: Supplementary file 1 — (DOCX 35 kb). [file 12265_2021_10111_MOESM1_ESM.docx]

**Supplemental Table 1. Recording and patient characteristics**

| no. | RA location | Rhythm | Gender | Age | Heart disease | History of AF |
| --- | --- | --- | --- | --- | --- | --- |
| 1 | mid | SR | M | 71 | CABG | no |
| 2 | superior | SR | F | 54 | CABG | no |
| 3 | superior | SR | M | 53 | CABG | paroxysmal |
| 4 | mid | Pacing (CL 250 ms) | M | 85 | CABG | paroxysmal |
| 5 | superior | AES | F | 69 | AVD | no |
| 6 | superior | SR | F | 50 | CABG | no |
| 7 | superior | SR | M | 67 | CABG | no |
| 8 | superior | AES | M | 68 | CABG, MVD | paroxysmal |
| 9 | superior | AES | M | 66 | CABG, AVD | paroxysmal |
| 10 | superior | SR | M | 61 | CABG | no |
| 11 | superior | SR | M | 55 | CABG | no |
| 12 | mid | SR | M | 63 | CABG | no |
| 13 | left atrium | AES | M | 72 | MVD | paroxysmal |
| 14 | inferior | AES | M | 72 | CABG, MVD | no |
| 15 | superior | AES | F | 48 | MVD | paroxysmal |
| 16 | mid | SR | F | 74 | MVD, TVD | persistent |
| 17 | superior | SR | F | 67 | MVD | paroxysmal |
| 18 | mid | AES | M | 58 | CABG, AVD | no |
| 19 | superior | SR | M | 58 | AVD, MVD | no |
| 20 | superior | SR | F | 72 | AVD, MVD, TVD | paroxysmal |
| 21 | mid | SR | M | 71 | CABG, MVD | paroxysmal |
| 22 | superior | SR | M | 67 | CABG | no |
| RA = right atrium, SR = sinus rhythm, CL = cycle length, AES = atrial extrasystole, M = male, F = female, CABG = coronary artery bypass grafting, AVD = aortic valve disease, MVD = mitral valve disease, TVD = tricuspid valve disease, AF = atrial fibrillation. | | | | | | |

Supplemental Table 2. Percentage of electrograms with EEA corresponding fractionation per patient

|  | **Observer 1** | | | | | | **Observer 2** | | | | | |
| --- | --- | --- | --- | --- | --- | --- | --- | --- | --- | --- | --- | --- |
|  | Epicardium | | | Endocardium | | | Epicardium | | | Endocardium | | |
| no. | uni | bi-x | bi-y | uni | bi-x | bi-y | uni | bi-x | bi-y | uni | bi-x | bi-y |
| 1 | 75 | 78 | 92 | 50 | 89 | 77 | 75 | 67 | 92 | 25 | 89 | 77 |
| 2 | 100 | 100 | 75 | 88 | 100 | 38 | 88 | 100 | 25 | 100 | 100 | 38 |
| 3 | 56 | 95 | 67 | 70 | 71 | 87 | 56 | 81 | 63 | 70 | 62 | 77 |
| 4 | 67 | 85 | 100 | 89 | 100 | 100 | 100 | 54 | 88 | 100 | 92 | 50 |
| 5 | 73 | 22 | 43 | 34 | 33 | 74 | 32 | 13 | 15 | 18 | 22 | 66 |
| 6 | 11 | 85 | 23 | 100 | 100 | 100 | 44 | 69 | 38 | 100 | 100 | 100 |
| 7 | 20 | 86 | 14 | 100 | 100 | 100 | 40 | 71 | 14 | 100 | 86 | 100 |
| 8 | 100 | 100 | 50 | 86 | 73 | 60 | 100 | 45 | 60 | 71 | 73 | 50 |
| 9 | 40 | 83 | 75 | 100 | 100 | 100 | 60 | 83 | 63 | 100 | 100 | 100 |
| 10 | 33 | 100 | 100 | 17 | 80 | 0 | 33 | 90 | 100 | 0 | 80 | 0 |
| 11 | 3 | 44 | 25 | 73 | 54 | 68 | 3 | 38 | 20 | 70 | 26 | 30 |
| 12 | 100 | 50 | 88 | 100 | 100 | 88 | 86 | 25 | 88 | 100 | 100 | 88 |
| 13 | 100 | 100 | 100 | 89 | 13 | 92 | 100 | 100 | 100 | 89 | 25 | 92 |
| 14 | 16 | 16 | 22 | 60 | 11 | 4 | 16 | 16 | 22 | 0 | 5 | 4 |
| 15 | 100 | 78 | 100 | 0 | 89 | 0 | 86 | 22 | 100 | 0 | 100 | 0 |
| 16 | 67 | 50 | 86 | 100 | 75 | 100 | 89 | 25 | 64 | 100 | 75 | 93 |
| 17 | 0 | 4 | 0 | 67 | 75 | 87 | 0 | 8 | 4 | 67 | 67 | 87 |
| 18 | 65 | 0 | 80 | 88 | 50 | 87 | 65 | 0 | 27 | 82 | 50 | 87 |
| 19 | 100 | 100 | 100 | 0 | 100 | 0 | 100 | 100 | 100 | 17 | 50 | 0 |
| 20 | 91 | 48 | 86 | 70 | 97 | 73 | 91 | 30 | 82 | 70 | 82 | 68 |
| 21 | 97 | 20 | 20 | 72 | 14 | 28 | 95 | 18 | 12 | 74 | 22 | 28 |
| 22 | 86 | 60 | 71 | 71 | 80 | 57 | 86 | 55 | 79 | 64 | 75 | 36 |
| median | 70 | 78 | 75 | 73 | 80 | 76 | 80 | 50 | 63 | 71 | 75 | 67 |

Supplemental Table 3. Voltage and relative voltage of corresponding fractionation per patient

|  | **Observer 1** | | | | | | | | | | | | **Observer 2** | | | | | | | | | | | |
| --- | --- | --- | --- | --- | --- | --- | --- | --- | --- | --- | --- | --- | --- | --- | --- | --- | --- | --- | --- | --- | --- | --- | --- | --- |
|  | Voltage (mV) | | | | | | Relative Voltage (%) | | | | | | Voltage (mV) | | | | | | Relative Voltage (%) | | | | | |
|  | Epicardium | | | Endocardium | | | Epicardium | | | Endocardium | | | Epicardium | | | Endocardium | | | Epicardium | | | Endocardium | | |
| no. | uni | bi-x | bi-y | uni | bi-x | bi-y | uni | bi-x | bi-y | uni | bi-x | bi-y | uni | bi-x | bi-y | uni | bi-x | bi-y | uni | bi-x | bi-y | uni | bi-x | bi-y |
| 1 | 1.27 | 0.29 | 0.96 | 1.60 | 0.33 | 0.60 | 23 | 4 | 26 | 30 | 7 | 8 | 1.24 | 0.31 | 1.04 | 2.15 | 0.32 | 0.63 | 23 | 5 | 30 | 54 | 9 | 10 |
| 2 | 0.95 | 0.23 | 0.20 | 0.29 | 0.09 | 0.09 | 25 | 22 | 13 | 31 | 69 | 50 | 1.15 | 0.22 | 0.30 | 0.27 | 0.09 | 0.10 | 29 | 21 | 27 | 27 | 65 | 71 |
| 3 | 0.69 | 0.31 | 0.25 | 1.64 | 0.36 | 0.30 | 35 | 40 | 20 | 44 | 32 | 19 | 0.57 | 0.30 | 0.25 | 1.74 | 0.37 | 0.37 | 29 | 39 | 20 | 45 | 35 | 22 |
| 4 | 0.63 | 0.18 | 0.30 | 0.56 | 0.17 | 0.18 | 21 | 10 | 21 | 35 | 49 | 16 | 0.56 | 0.19 | 0.32 | 0.57 | 0.20 | 0.18 | 13 | 9 | 21 | 35 | 58 | 18 |
| 5 | 0.41 | 0.15 | 0.10 | 0.46 | 0.11 | 0.13 | 28 | 26 | 12 | 77 | 42 | 19 | 0.45 | 0.10 | 0.06 | 1.24 | 0.17 | 0.13 | 58 | 39 | 17 | 70 | 48 | 35 |
| 6 | 0.23 | 0.07 | 0.15 | 1.57 | 0.32 | 0.48 | 25 | 41 | 40 | 172 | 38 | 64 | 0.15 | 0.11 | 0.18 | 1.57 | 0.30 | 0.52 | 12 | 47 | 28 | 172 | 38 | 65 |
| 7 | 0.33 | 0.48 | 1.15 | 1.99 | 0.52 | 0.48 | 2 | 12 | 62 | 107 | 29 | 27 | 0.17 | 0.57 | 1.14 | 1.99 | 0.40 | 0.58 | 2 | 10 | 61 | 107 | 26 | 25 |
| 8 | 1.42 | 0.06 | 0.61 | 0.12 | 0.07 | 0.14 | 44 | 14 | 41 | 10 | 23 | 35 | 1.38 | 0.09 | 0.41 | 0.17 | 0.08 | 0.17 | 44 | 19 | 43 | 14 | 21 | 70 |
| 9 | 0.19 | 0.39 | 0.50 | 1.77 | 0.48 | 0.49 | 6 | 50 | 31 | 78 | 38 | 34 | 0.16 | 0.37 | 0.68 | 1.77 | 0.51 | 0.45 | 3 | 58 | 37 | 78 | 40 | 29 |
| 10 | 2.28 | 0.76 | 0.77 | 0.23 | 0.17 | - | 107 | 25 | 28 | 21 | 41 | - | 2.28 | 0.79 | 0.71 | - | 0.20 | - | 107 | 24 | 21 | - | 71 | - |
| 11 | 0.25 | 0.17 | 0.23 | 0.51 | 0.09 | 0.12 | 9 | 9 | 11 | 19 | 7 | 9 | 0.25 | 0.19 | 0.33 | 0.46 | 0.09 | 0.14 | 9 | 14 | 15 | 18 | 7 | 26 |
| 12 | 0.94 | 0.09 | 0.16 | 0.66 | 0.28 | 0.17 | 27 | 8 | 19 | 36 | 63 | 24 | 0.92 | 0.06 | 0.17 | 0.66 | 0.25 | 0.27 | 31 | 6 | 15 | 36 | 58 | 31 |
| 13 | 1.55 | 0.30 | 0.42 | 1.12 | 0.40 | 0.25 | 16 | 6 | 15 | 40 | 90 | 23 | 1.55 | 0.29 | 0.49 | 1.12 | 0.61 | 0.21 | 16 | 6 | 17 | 40 | 87 | 25 |
| 14 | 0.21 | 0.12 | 0.09 | 0.18 | 0.03 | 0.07 | 13 | 60 | 32 | 50 | 56 | 74 | 0.20 | 0.14 | 0.10 | - | 0.04 | 0.07 | 12 | 73 | 43 | - | 80 | 59 |
| 15 | 0.59 | 0.08 | 0.33 | - | 0.05 | - | 37 | 10 | 34 | - | 69 | - | 0.67 | 0.29 | 0.41 | - | 0.05 | - | 39 | 18 | 50 | - | 73 | - |
| 16 | 0.07 | 0.04 | 0.08 | 0.27 | 0.04 | 0.06 | 13 | 32 | 32 | 66 | 40 | 41 | 0.06 | 0.04 | 0.12 | 0.27 | 0.04 | 0.07 | 13 | 44 | 49 | 66 | 39 | 50 |
| 17 | - | 0.19 | - | 2.27 | 0.23 | 0.31 | - | 59 | - | 163 | 27 | 40 | - | 0.15 | 0.11 | 2.29 | 0.34 | 0.29 | - | 45 | 30 | 163 | 35 | 38 |
| 18 | 0.73 | - | 0.16 | 0.44 | 0.10 | 0.16 | 16 | - | 5 | 49 | 33 | 63 | 0.62 | - | 0.18 | 0.45 | 0.12 | 0.11 | 14 | - | 7 | 48 | 44 | 50 |
| 19 | 5.91 | 0.85 | 0.14 | - | 0.51 | - | 84 | 19 | 2 | - | 34 | - | 5.91 | 0.84 | 0.17 | 0.07 | 0.62 | - | 84 | 18 | 3 | 1 | 39 |  |
| 20 | 0.43 | 0.26 | 0.36 | 1.55 | 0.05 | 0.24 | 7 | 10 | 20 | 168 | 27 | 42 | 0.43 | 0.19 | 0.29 | 1.55 | 0.13 | 0.20 | 7 | 24 | 25 | 168 | 30 | 46 |
| 21 | 0.39 | 0.04 | 0.09 | 0.32 | 0.05 | 0.06 | 18 | 7 | 20 | 54 | 34 | 48 | 0.38 | 0.04 | 0.12 | 0.30 | 0.05 | 0.10 | 17 | 17 | 26 | 53 | 46 | 66 |
| 22 | 2.12 | 0.20 | 0.43 | 0.21 | 0.61 | 0.64 | 68 | 21 | 19 | 8 | 34 | 27 | 2.12 | 0.22 | 0.44 | 0.22 | 0.58 | 0.67 | 68 | 20 | 15 | 9 | 34 | 33 |
| median | 0.63 | 0.19 | 0.25 | 0.54 | 0.17 | 0.18 | 23 | 19 | 20 | 47 | 36 | 34 | 0.57 | 0.19 | 0.29 | 0.66 | 0.20 | 0.20 | 17 | 20 | 25 | 48 | 40 | 35 |

Supplemental Table 4. SNR and time accuracy of corresponding fractionation per patient

|  | **Observer 1** | | | | | | | | | | | | **Observer 2** | | | | | | | | | | | |
| --- | --- | --- | --- | --- | --- | --- | --- | --- | --- | --- | --- | --- | --- | --- | --- | --- | --- | --- | --- | --- | --- | --- | --- | --- |
|  | SNR | | | | | | Time accuracy (ms) | | | | | | SNR | | | | | | Time accuracy (ms) | | | | | |
|  | Epicardium | | | Endocardium | | | Epicardium | | | Endocardium | | | Epicardium | | | Endocardium | | | Epicardium | | | Endocardium | | |
| no. | uni | bi-x | bi-y | uni | bi-x | bi-y | uni | bi-x | bi-y | uni | bi-x | bi-y | uni | bi-x | bi-y | uni | bi-x | bi-y | uni | bi-x | bi-y | uni | bi-x | bi-y |
| 1 | 31 | 13 | 20 | 28 | 17 | 11 | 2 | 3 | 1 | 5 | 4 | 3 | 31 | 16 | 23 | 45 | 15 | 12 | 2 | 3 | 1 | 4 | 3 | 3 |
| 2 | 22 | 8 | 4 | 8 | 4 | 2 | 0 | 1 | 2 | 1 | 0 | 4 | 24 | 8 | 6 | 7 | 3 | 3 | 0 | 1 | 1 | 1 | 0 | 4 |
| 3 | 13 | 19 | 4 | 25 | 15 | 4 | 1 | 2 | 4 | 3 | 2 | 3 | 10 | 19 | 4 | 26 | 19 | 4 | 1 | 1 | 3 | 3 | 1 | 3 |
| 4 | 5 | 8 | 4 | 6 | 9 | 3 | 4 | 2 | 3 | 4 | 3 | 3 | 5 | 8 | 4 | 6 | 11 | 3 | 3 | 3 | 3 | 3 | 3 | 4 |
| 5 | 17 | 12 | 6 | 20 | 10 | 7 | 1 | 5 | 2 | 2 | 5 | 3 | 20 | 9 | 3 | 60 | 13 | 7 | 0 | 5 | 1 | 3 | 5 | 3 |
| 6 | 3 | 3 | 3 | 16 | 14 | 9 | 4 | 3 | 2 | 0 | 1 | 0 | 2 | 5 | 4 | 16 | 13 | 9 | 3 | 2 | 5 | 0 | 1 | 0 |
| 7 | 3 | 12 | 17 | 15 | 15 | 10 | 0 | 4 | 4 | 0 | 1 | 1 | 1 | 15 | 16 | 15 | 11 | 10 | 3 | 4 | 4 | 0 | 2 | 1 |
| 8 | 20 | 2 | 10 | 2 | 2 | 2 | 1 | 3 | 2 | 1 | 1 | 2 | 20 | 3 | 7 | 2 | 2 | 2 | 1 | 3 | 2 | 1 | 1 | 2 |
| 9 | 3 | 13 | 14 | 34 | 17 | 11 | 5 | 2 | 3 | 4 | 2 | 1 | 3 | 14 | 17 | 34 | 18 | 10 | 4 | 2 | 2 | 4 | 2 | 1 |
| 10 | 12 | 21 | 4 | 2 | 4 | - | 5 | 3 | 3 | 2 | 4 | - | 12 | 20 | 4 | - | 5 | - | 5 | 3 | 3 | - | 3 | - |
| 11 | 8 | 10 | 7 | 17 | 4 | 3 | 6 | 5 | 3 | 5 | 5 | 3 | 8 | 11 | 9 | 15 | 4 | 4 | 6 | 6 | 5 | 5 | 5 | 5 |
| 12 | 13 | 2 | 3 | 10 | 6 | 3 | 2 | 2 | 3 | 2 | 2 | 2 | 13 | 1 | 3 | 10 | 6 | 5 | 3 | 3 | 3 | 2 | 2 | 2 |
| 13 | 16 | 12 | 6 | 10 | 12 | 2 | 3 | 1 | 2 | 2 | 1 | 3 | 16 | 12 | 7 | 10 | 18 | 2 | 3 | 1 | 2 | 2 | 4 | 3 |
| 14 | 3 | 4 | 2 | 3 | 1 | 1 | 1 | 6 | 4 | 2 | 4 | 2 | 3 | 5 | 2 | - | 2 | 1 | 1 | 6 | 4 | - | 6 | 2 |
| 15 | 30 | 9 | 18 | - | 4 | - | 4 | 2 | 3 | - | 2 | - | 32 | 27 | 23 | - | 4 | - | 4 | 2 | 4 | - | 2 | - |
| 16 | 2 | 4 | 2 | 7 | 3 | 2 | 2 | 3 | 0 | 4 | 1 | 3 | 1 | 4 | 4 | 7 | 3 | 2 | 3 | 3 | 0 | 4 | 1 | 4 |
| 17 | - | 7 | - | 47 | 14 | 5 | - | 1 | - | 5 | 2 | 3 | - | 7 | 2 | 47 | 27 | 5 | - | 2 | 2 | 5 | 2 | 3 |
| 18 | 28 | - | 5 | 11 | 5 | 5 | 0 | - | 3 | 3 | 2 | 2 | 22 | - | 7 | 10 | 6 | 3 | 0 | - | 4 | 3 | 2 | 2 |
| 19 | 202 | 71 | 4 | - | 47 | - | 3 | 3 | 3 | - | 1 | - | 202 | 69 | 7 | 5 | 86 | - | 3 | 3 | 2 | 0 | 1 | - |
| 20 | 9 | 21 | 13 | 23 | 4 | 6 | 3 | 3 | 2 | 3 | 3 | 4 | 9 | 16 | 11 | 23 | 10 | 4 | 3 | 2 | 2 | 3 | 3 | 4 |
| 21 | 5 | 1 | 2 | 4 | 1 | 1 | 1 | 4 | 6 | 4 | 3 | 4 | 5 | 1 | 2 | 4 | 1 | 2 | 1 | 5 | 6 | 4 | 3 | 5 |
| 22 | 33 | 10 | 5 | 3 | 23 | 7 | 1 | 2 | 1 | 1 | 2 | 2 | 33 | 9 | 4 | 3 | 24 | 7 | 1 | 2 | 2 | 1 | 1 | 3 |
| median | 13 | 10 | 5 | 11 | 8 | 4 | 2 | 3 | 3 | 3 | 2 | 3 | 12 | 9 | 5 | 10 | 10 | 4 | 3 | 3 | 3 | 3 | 2 | 3 |

Supplemental Table 5. Percentage of electrograms with additional fractionation per patient

|  | **Observer 1** | | | | | | **Observer 2** | | | | | |
| --- | --- | --- | --- | --- | --- | --- | --- | --- | --- | --- | --- | --- |
|  | Epicardium | | | Endocardium | | | Epicardium | | | Endocardium | | |
| no. | uni | bi-x | bi-y | uni | bi-x | bi-y | uni | bi-x | bi-y | uni | bi-x | bi-y |
| 1 | 100 | 100 | 100 | 75 | 100 | 77 | 100 | 100 | 100 | 75 | 89 | 77 |
| 2 | 0 | 100 | 25 | 0 | 100 | 50 | 6 | 100 | 25 | 0 | 100 | 100 |
| 3 | 33 | 71 | 73 | 56 | 71 | 87 | 30 | 48 | 70 | 52 | 76 | 67 |
| 4 | 0 | 8 | 63 | 0 | 69 | 38 | 0 | 8 | 38 | 0 | 46 | 25 |
| 5 | 84 | 72 | 91 | 93 | 94 | 98 | 45 | 76 | 77 | 84 | 94 | 98 |
| 6 | 0 | 62 | 31 | 11 | 92 | 92 | 0 | 62 | 77 | 11 | 100 | 77 |
| 7 | 60 | 43 | 71 | 80 | 57 | 100 | 60 | 14 | 14 | 80 | 57 | 86 |
| 8 | 100 | 100 | 100 | 95 | 100 | 60 | 95 | 100 | 90 | 95 | 100 | 50 |
| 9 | 60 | 100 | 75 | 0 | 100 | 88 | 80 | 100 | 75 | 0 | 100 | 88 |
| 10 | 50 | 50 | 56 | 92 | 80 | 0 | 50 | 50 | 67 | 33 | 80 | 22 |
| 11 | 3 | 23 | 8 | 63 | 77 | 78 | 3 | 21 | 3 | 57 | 77 | 63 |
| 12 | 100 | 0 | 38 | 100 | 25 | 50 | 100 | 0 | 50 | 100 | 25 | 63 |
| 13 | 0 | 25 | 8 | 11 | 38 | 83 | 0 | 25 | 17 | 11 | 0 | 83 |
| 14 | 40 | 32 | 63 | 92 | 5 | 0 | 52 | 32 | 52 | 88 | 26 | 4 |
| 15 | 86 | 22 | 100 | 0 | 67 | 50 | 57 | 11 | 50 | 0 | 67 | 50 |
| 16 | 78 | 100 | 71 | 89 | 100 | 21 | 78 | 100 | 79 | 78 | 100 | 29 |
| 17 | 0 | 25 | 4 | 47 | 96 | 83 | 0 | 29 | 9 | 40 | 92 | 83 |
| 18 | 35 | 0 | 20 | 88 | 100 | 87 | 12 | 0 | 0 | 88 | 100 | 60 |
| 19 | 0 | 100 | 100 | 33 | 50 | 50 | 0 | 100 | 100 | 0 | 50 | 0 |
| 20 | 9 | 67 | 50 | 22 | 91 | 36 | 9 | 36 | 45 | 22 | 58 | 55 |
| 21 | 38 | 80 | 68 | 28 | 33 | 20 | 62 | 61 | 68 | 26 | 35 | 56 |
| 22 | 57 | 65 | 86 | 71 | 90 | 93 | 50 | 55 | 71 | 71 | 85 | 64 |
| median | 39 | 63 | 65 | 59 | 85 | 68 | 48 | 49 | 59 | 46 | 78 | 63 |

Supplemental Table 6. Average number of additional fractionation per electrogram per patient

|  | **Observer 1** | | | | | | **Observer 2** | | | | | |
| --- | --- | --- | --- | --- | --- | --- | --- | --- | --- | --- | --- | --- |
|  | Epicardium | | | Endocardium | | | Epicardium | | | Endocardium | | |
| no. | uni | bi-x | bi-y | uni | bi-x | bi-y | uni | bi-x | bi-y | uni | bi-x | bi-y |
| 1 | 1 | 3 | 4 | 1 | 2 | 2 | 1 | 3 | 4 | 1 | 2 | 1 |
| 2 | 0 | 2 | 1 | 0 | 1 | 1 | 0 | 2 | 1 | 0 | 3 | 1 |
| 3 | 0 | 2 | 1 | 1 | 2 | 2 | 0 | 2 | 1 | 1 | 2 | 1 |
| 4 | 0 | 0 | 1 | 0 | 1 | 0 | 0 | 0 | 1 | 0 | 1 | 0 |
| 5 | 1 | 3 | 3 | 2 | 5 | 5 | 1 | 2 | 2 | 1 | 4 | 4 |
| 6 | 0 | 1 | 0 | 0 | 2 | 1 | 0 | 1 | 1 | 0 | 3 | 2 |
| 7 | 1 | 1 | 1 | 1 | 1 | 3 | 1 | 0 | 0 | 1 | 1 | 2 |
| 8 | 1 | 2 | 1 | 2 | 3 | 1 | 1 | 1 | 1 | 1 | 3 | 1 |
| 9 | 1 | 3 | 1 | 0 | 2 | 3 | 1 | 3 | 2 | 0 | 3 | 2 |
| 10 | 1 | 1 | 1 | 1 | 1 | 0 | 1 | 1 | 1 | 0 | 1 | 0 |
| 11 | 0 | 0 | 0 | 1 | 2 | 2 | 0 | 0 | 0 | 1 | 2 | 1 |
| 12 | 2 | 0 | 0 | 2 | 0 | 1 | 2 | 0 | 1 | 2 | 0 | 1 |
| 13 | 0 | 0 | 0 | 0 | 0 | 1 | 0 | 0 | 0 | 0 | 0 | 1 |
| 14 | 0 | 0 | 1 | 1 | 0 | 0 | 1 | 0 | 1 | 1 | 0 | 0 |
| 15 | 1 | 0 | 2 | 0 | 1 | 1 | 1 | 0 | 1 | 0 | 1 | 1 |
| 16 | 1 | 3 | 1 | 1 | 2 | 0 | 1 | 3 | 1 | 1 | 2 | 0 |
| 17 | 0 | 1 | 0 | 1 | 4 | 2 | 0 | 1 | 0 | 1 | 3 | 3 |
| 18 | 0 | 0 | 0 | 1 | 2 | 2 | 0 | 0 | 0 | 1 | 3 | 1 |
| 19 | 0 | 2 | 3 | 0 | 1 | 1 | 0 | 2 | 3 | 0 | 1 | 0 |
| 20 | 0 | 1 | 1 | 0 | 1 | 0 | 0 | 0 | 1 | 0 | 1 | 1 |
| 21 | 0 | 1 | 1 | 0 | 0 | 0 | 1 | 1 | 1 | 0 | 0 | 1 |
| 22 | 1 | 2 | 2 | 1 | 3 | 2 | 1 | 2 | 2 | 1 | 3 | 1 |
| median | 0 | 1 | 1 | 1 | 2 | 1 | 1 | 1 | 1 | 1 | 2 | 1 |
